# Assessment of patient-reported outcome measures in the surgical treatment of patients with gastric cancer

**DOI:** 10.1007/s00464-015-4415-3

**Published:** 2015-08-27

**Authors:** Jennifer Straatman, Nicole van der Wielen, Pieter J. Joosten, Caroline B. Terwee, Miguel A. Cuesta, Elise P. Jansma, Donald L. van der Peet

**Affiliations:** Department of Gastrointestinal Surgery, VU University Medical Center, De Boelelaan 1117, ZH 7F020, 1081 HV Amsterdam, The Netherlands; Department of Epidemiology and Biostatistics, EMGO Institute for Health and Care Research, VU University Medical Center, Amsterdam, The Netherlands; Medical Library, VU University Medical Center, Amsterdam, The Netherlands

**Keywords:** Quality of life, Gastric cancer, Gastrectomy, PROMs

## Abstract

**Background:**

Gastric cancer is responsible for 10 % of all cancer-related deaths worldwide. With improved operative techniques and neo-adjuvant therapy, survival rates are increasing. Outcomes of interest are shifting to quality of life (QOL), with many different tools available. The aim of this study was to assess which patient-reported outcome measures (PROMs) are used to measure QOL after a gastrectomy for cancer.

**Methods:**

A comprehensive search was conducted for original articles investigating QOL after gastrectomy. Two authors independently selected relevant articles, conducted clinical appraisal and extracted data (P.J. and J.S.).

**Results:**

Out of 3414 articles, 26 studies were included, including a total of 4690 patients. These studies included ten different PROMs, which could be divided into generic, symptom-specific and disease-specific questionnaires. The EORTC and the FACT questionnaires use an oncological overall QOL module and an organ-specific module. Only one validation study regarding the use of the EORTC after surgery for gastric cancer was available, demonstrating good psychometric properties and clinical validity.

**Conclusions:**

A great variety of PROMs are being used in the measurement of QOL after surgery for gastric cancer. A questionnaire with a general module along with a disease-specific module for the assessment of QOL seems most desirable, such as the EORTC and the FACT with their specific modules. Both are developed in different treatment modalities, such as in surgical patients. EORTC is the most widely used questionnaire and therefore allows for comparison of new studies to existing data. Future studies are needed to assess content validity in surgical gastric cancer patients.

Gastric cancer is responsible for 10 % of all cancer-related deaths worldwide, with the highest incidences in Eastern Asia, Eastern Europe and South America [1]. Although multiple treatment modalities exist, surgical resection of the primary tumour and regional lymph nodes is still the only curative treatment available for gastric cancer [2]. Currently, the 5-year survival rate after oesophageal resection is approximately 20 % [3]. With the implementation of minimally invasive techniques and additional treatments such as neo-adjuvant chemotherapy, survival rates have improved and an according number of long-term survivors exists [4–6]. Laparoscopic techniques have been shown to improve quality of life sooner after surgery [7].

With increasing survival and decreased morbidity, a shift in interest of outcome parameters is seen from survival and morbidity rates to the impact of radical gastrectomy and chemoradiotherapy on patient-reported outcomes, such as quality of life (QOL) [8]. Information about QOL outcomes should be an important outcome parameter in research regarding the optimal treatment for gastric cancer.

The World Health Organization (WHO) defined QOL as an individuals’ perception of their position in life in the cultural context and in the value system in which they live and in relation to their goals, expectations, standards and concerns [9]. QOL data provide direct measures of benefit as perceived by the patient and may be useful in clarifying treatment preferences. Many different questionnaires are available, both validated and non-validated, to assess the quality of life [7]. Although the different instruments focus on different aspects of QOL, no consensus exists as to which instrument is optimal in the assessment of QOL after gastrectomy for gastric cancer [10]. The aim of this systematic review was to assess which PROMs are used in the assessment of QOL after surgery for gastric cancer.

## Materials and methods

### Literature search

To identify all relevant publications, a systematic search in the bibliographic databases PubMed, EMBASE and The Cochrane Library (via Wiley) from inception to 14 October 2014 was performed. Search terms included controlled terms from MeSH in PubMed, Emtree in EMBASE.com as well as free text terms. Free text terms were only used in The Cochrane library. Search terms expressing “stomach neoplasm” were used in combination with search terms comprising “surgery”. Moreover, an extensive search filter for finding patient-reported outcome measures was used, developed by the University of Oxford (“Appendix”). The reference list of included articles was hand-searched for relevant publications.

### Selection criteria and definitions

Two authors (P.J. and J.S.) independently evaluated the search findings for potential eligibility for systematic review using the MEDLINE, EMBASE and Cochrane databases. The inclusion criteria were: (1) article published in English language; (2) only full-text articles, no abstracts or case reports were included and (3) the study had to investigate QOL after gastric resection using questionnaires (i.e. non-structured interviews were not included). (4) Only patients with gastric carcinoma were included. Studies that described gastrointestinal stromal tumours (GIST) and benign tumours were excluded. Distal, proximal, subtotal and total gastrectomies were included. Wedge resections and local resections were excluded. Regarding surgical techniques, both open and minimally invasive procedures were included, and various reconstructive methods were included (i.e. Roux-en Y or Billroth reconstruction).

### Data extraction and quality assessment

The reviewers (P.J. and J.S.) extracted the following data from each study: first author, title of the article, year of publication, type of study, type of gastrectomy, type of reconstruction, number of patients included and the PROMs used to assess QOL. All articles that were deemed suitable after full-text analysis were assessed for quality of the performed study.

## Results

### Study selection

**Fig. 1:**
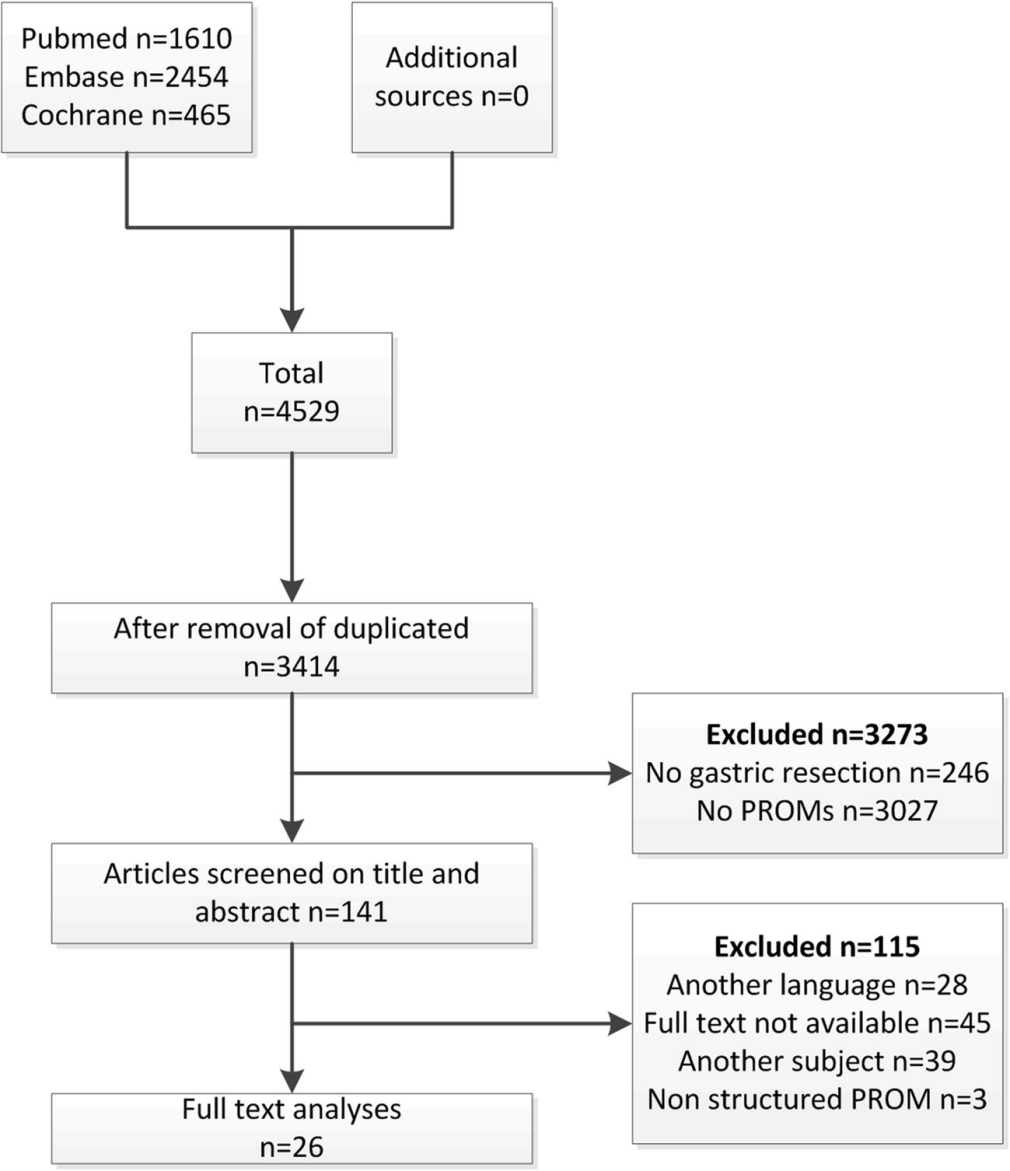
Flow chart for the selection of articles for systematic review

Initially, the literature search of MEDLINE, EMBASE and Cochrane resulted in 4529 hits, after removal of duplicates 3414 hits remained. The articles were screened based on title and abstract by two different authors (P.J. and J.S.) independently, and this resulted in a selection of 141 articles for full-text analysis. Of these 141 articles, another 115 were excluded since they did not meet the predefined criteria as described in the methods section; 28 articles were published in another language than English; 45 references consisted only of conference abstracts; 39 articles included a different subject; a final three articles were excluded because they did not use questionnaires but self-reported interviews for QOL assessment. Twenty-six articles remained for further analysis. A flow chart of the article selection is depicted in Fig. 1.

### Study characteristics

Twenty-six articles were included for full-text analysis, of which twelve articles were prospective cohort studies, six of which were randomized controlled trials, and fourteen were retrospective cohort studies with prospective QOL assessment, including a total of 4690 patients. One study was a development and validation study [11]. There was great dispersion in follow-up data, ranging from 6 months to 5 years. An overview of the included articles is given in Table 1 for prospective articles and Table 2 for retrospective studies.

**Table 1:** Description of prospective cohort studies

Study	Country	Study type	Patient (*n*)	Aim	QOL instruments	Follow-up
Zieren et al. [31]	Germany	Prospective	106	Long-term follow-up after gastrectomy	EORTC QLQ-C36 Spitzer index	12 months
Wu et al. [12]	Taiwan	Prospective, RCT	214	D1 versus D3 lymphadenectomy	Spitzer index	Baseline, 6, 12, 24, 36, 48 and 60 months
Avery et al. [32]	UK	Prospective	58	QOL in patients that died within 2 years versus survivors	EORTC QLQ-C30 EORTC QLQ-STO22	Baseline, 3,6,9, and 12, 18 and 24 months
Svedlund et al. [17]	Sweden	Prospective, RCT	64	Total or subtotal gastrectomy, with or without pouch reconstruction	GSRS SIP	Baseline, 3,12, 24, 36, 48 and 60 months
Karanicolas et al. [33]	USA	Prospective	134	Total, distal or proximal gastrectomy	EORTC QLQ-C30 EORTC QLQ-STO22	Baseline, 3, 6, 9, 12, 18 months
Munene et al. [21]	Canada	Prospective	43	Partial versus total gastrectomy	FACT-G FACT-GA	Baseline, every 3 months in 2 years
Kim et al. [34]	Korea	Prospective	465	Total versus subtotal gastrectomy	EORTC QLQ-C30 EORTC QLQ-STO22	Baseline, 3 and 12 months
Takiguchi et al. [29]	Japan	Prospective, RCT	268	Roux-en Y versus Billroth I reconstruction	EORTC QLQ-C30 DAUGS20	21 months (range 3 - 34)
Kono et al. [18]	Japan	Prospective, RCT	47	Roux-en Y versus pouch reconstruction	GSRS	3, 12, 48 months
Horvath et al. [16]	Hungary	Prospective, RCT	46	Roux-en Y versus pouch reconstruction	GIQLI	6, 12 and 24 months
Scurtu et al. [25]	Romania	Prospective	39	Total gastrectomy with E–E versus E–S anastomosis	Korenaga score	3 and 12 months
Kim et al. [35]	Korea	Prospective, RCT	164	Open versus laparoscopy-assisted distal gastrectomy	EORTC QLQ-C30 EORTC QLQ-STO22	Baseline, 1, 3, 6 and 12 months

**Table 2:** Description of retrospective cohort studies

Study	Country	Study type	Patient (*n*)	Aim	QOL instruments	Follow-up
Amemiya et al. [36]	Japan	Retrospective	223	Patients older than 75 years	SF-12 EQ-5D	Baseline, 1,3 and 6 months
Rausei et al. [37]	Italy	Retrospective	103	Total versus subtotal resection, lymphadenectomy and multivisceral resection	EORTC QLQ-C30 EORTC QLQ-STO22	Mean follow-up 81 ± 80.7 months
Park et al. [38]	Korea	Retrospective	275	Total versus subtotal/distal resection	EORTC QLQ-C30 EORTC QLQ-STO22	Baseline, 3, 6, 9, 12, 18 and 24 months
Díaz de Liaño et al. [39]	Spain	Retrospective	54	Total versus subtotal gastrectomy and D1 versus D2 lymphadenectomy	EORTC QLQ-C30	49 months (range 41–89)
Buhl et al. [40]	Germany	Retrospective	104	Distal versus total gastrectomy with Roux-en Y or pouch	Spitzer index	12 months
Bae et al. [41]	Korea	Retrospective	391	Total versus subtotal	EORTC QLQ-C30 EORTC QLQ-STO22	27.5 ± 3.3 months
Huang et al. [42]	Taiwan	Retrospective	51	Total versus subtotal gastrectomy, early versus late stage	EORTC QLQ-C30 EORTC QLQ-STO22	17 months (range 6–24 months)
Soo Lee et al. [43]	Korea	Retrospective	80	Open versus laparoscopy-assisted distal gastrectomy	EORTC QLQ-C30 EORTC QLQ-STO22	6 months to 5 year range
Tyrvainen et al. [44]	Finland	Retrospective	172	QOL in long-term survivors after total gastrectomy	SF-36 15D	Median 9 (6–19) years
Nakamura et al. [11]	Japan	Retrospective	883	Development and validation of DAUGS	DAUGS20	3 and 6 months, 1, 2 and 3 years
Nakamura et al. [45]	Japan	Retrospective	165	Evaluate DAUGS in patients after gastric resection	DAUGS32	3–6 months, 6–1 year, 1–2 years, 1–3 years
Kong et al. [46]	Korea	Retrospective	272	Chronological change of QOL after gastrectomy	EORTC QLQ-C30 EORTC QLQ-STO22	Baseline, 3, 6, 9 and 12 months
Soo Lee et al. [47]	Korea	Retrospective	143	QOL 5 years or more after total gastrectomy	EORTC QLQ-C30 EORTC QLQ-STO22	Mean 89.3 (range 66–201) months
Soo Lee et al. [48]	Korea	Retrospective	126	QOL of long-term survivors after distal subtotal gastrectomy	EORTC QLQ-C30 EORTC QLQ-STO22	5 years

### The quality-of-life instruments

Twenty-six full-text articles were assessed regarding QOL following surgical procedures for gastric cancer. In these articles, a total of ten different PROMs were described. Different instruments focussed on different dimensions of the QOL (i.e. physical, functional, social and emotional function).

The PROMs could be divided into separate categories, as given in Table 3. First four generic instruments were used, i.e. the Short Form-12 (SF-12), Sickness Impact Profile (SIP), Spitzer index and EuroQol-5D (EQ-5D). These instruments were used to compare results across different conditions of health. These questionnaires are developed and validated to measure QOL in a general population. The Spitzer index is a global health assessment tool, which assess activity, daily living, health, support system and outlook. No symptom- or treatment-specific questions are included in this questionnaire [12, 13]. The SF-12, SIP and EQ-5D have all been used once, and three out of the twenty studies have used the Spitzer index.

**Table 3:** Description of patient-reported outcome measures (PROMs)

	Questionnaires	Target population	Dimensions (number of items)	Ease of scoring and administration (range of scores)	Number of studies
Generic	SIP [49]	Very broad, tested in non-, in- and out-patient with different illnesses and different severities	Sleep and rest (7)	Easy (0–136)	1
			Eating (9)		
			Work (9)		
			Home management (10)		
			Recreation and pastimes (8)		
			Ambulation (10)		
			Mobility (10)		
			Body care and movement (23)		
			Social interaction (20)		
			Alertness behaviour (10)		
			Emotional behaviour (9)		
			Communication (9)		
			Total = 136		
	SF-12 [50]	General population	Physical functioning (2)	Easy (12–47)	1
			Role physical (2)		
			Bodily pain (1)		
			General health (1)		
			Vitality (1)		
			Social functioning (1)		
			Role emotional (2)		
			Mental health (2)		
	EQ-5D [51]	General population	Mobility	Easy (0–100 per dimension)	1
			Self-care		
			Usual activities		
			Pain/discomfort		
			Anxiety/depression		
	The Spitzer QOL index [13]	Cancer patients	Activity	Easy (0–2 per question) (0–10)	3
			Daily living		
			Health		
			Social support		
			Outlook		
Symptom focused	GIQLI [14, 52]	Developed in patients with benign or malignant disorders of the oesophagus, stomach, gallbladder, pancreas, small intestine, colon, and rectum. And developed in patients who underwent a laparoscopic chole-cystectomy	Physical well-being (10)	Easy (0–4 per question) (0–144)	1
			Mental well-being (5)		
			Gastrointestinal symptoms (16)		
			Single items (5)		
	GSRS [15, 53]	Developed for irritable bowel syndrome and peptic ulcer disease. Later validated in upper gastrointestinal patient	Abdominal pain syndrome	Easy (0–3 per question) (0–45)	2
			Reflux syndrome		
			Indigestion syndrome		
			Diarrhoea syndrome		
			Constipation syndrome		
Cancer specific	EORTC QLQ-C30 [22]	Cancer patients (developed in lung cancer patients)	Global health (2)	Easy (1–4 per question) (30–120)	15
			Functional scales		
			Physical (5)		
			Role (2)		
			Cognitive (2)		
			Emotional (4)		
			Social (2)		
			Symptom scales		
			Fatigue (3)		
			Pain (2)		
			Nausea and vomiting (2)		
			Single items (6)		
	FACT-G [19]	General cancer, developed in breast, lung and colorectal cancer	Physical (7)	Easy (1–4 per question) (0–108)	1
			Social/family (7)		
			Emotional (6)		
			Functional (7)		
Gastric cancer specific	EORTC QLQ-STO22 [23]	Patients with gastric cancer undergoing surgery, chemo- or chemoradiotherapy in curative or palliative setting	Five scales	Easy (1–4 per question) (22–88)	12
			Dysphagia (4)		
			Eating restrictions (5)		
			Pain (3)		
			Reflux (3)		
			Anxiety (3)		
			Three single items		
			Dry mouth (1)		
			Body image (1)		
			Hair loss (2)		
	FACT-Ga [20]	Gastric cancer (adenocarcinoma), gastrectomy, chemo and radiotherapy	Gastric cancer subscale (19)	Easy (0–4 per question) (0–76)	1
Postoperative	Korenaga’s score [25]	Treatment-specific after gastrectomy	Single items (14)	Easy (0–2 per question) (0–28)	1
	DAUGS20 [11]	Developed to assess postoperative dysfunction after surgery for gastric and oesophageal carcinoma	Single items (20)	Easy (1–5 per question) (34–170)	2
			Limited activity due to decreased food consumption		
			Reflux		
			Dumping		
			Nausea and vomiting		
			Deglutition difficulty		
			Pain		
			Difficulty in stool formation and passage		

*SIP* Sickness Impact Profile, *SF-12* The 12-item Short Form Healthy Survey, *EQ-5D* EuroQoL-5D, *GIQLI* Gastrointestinal Quality of Life Index, *GSRS* Gastrointestinal Symptom Rating Scale, *EORTC QLQ* European Organization for Research and Treatment QOL Questionnaire, *FACT-G* Functional Assessment of Cancer Therapy—General, *DAUGS* Dysfunction After Upper Gastrointestinal Surgery, *FACT-Ga* Functional Assessment of Cancer Therapy for patients with Gastric Cancer

Secondly, symptom-specific questionnaires were used, namely the Gastrontestinal Quality of Life Index (GIQLI) and the Gastrointestinal Symptom Rating Scale (GSRS). The GIQLI is developed in patients with benign and malignant disorders [14]. The GRSR was initially developed in patients with irritable bowel disease and not specifically designed for oncological or postoperative patients [15]. Only one study assessed QOL with the GIQLI score [16]. The GSRS score was used in two studies and allowed for overall assessment and of assessment of the individual items [17, 18]. GIQLI and GSRS are specifically designed for gastrointestinal symptoms, not for overall QOL.

A third group consists of disease-specific questionnaires. The Functional Assessment of Cancer Therapy (FACT) questionnaires consist of a general health module (FACT-G), and disease-specific modules can be added, such as FACT-Ga for gastric cancer [19, 20], thus allowing for the assessment of overall QOL and assessment of disease-specific symptoms by adding the appropriate module. The FACT-Ga is developed in patients with gastric cancer who underwent different treatment modalities, such as gastrectomy, chemotherapy and radiotherapy [20]. One study has used the FACT questionnaire [21].

The European Organisation for Research and Treatment of Cancer (EORTC) questionnaires work in a similar fashion, consisting of a general health questionnaire, the EORTC QLQ-C30, which is aimed specifically at cancer patients [22]. Disease-specific modules can be added, such as the EORTC QLQ-STO22 for gastric cancer. The EORTC QLQ-STO22 is developed in patients with gastric cancer who underwent different treatment modalities, such as surgery, chemo- or chemoradiotherapy in curative or palliative setting [23, 24]. The EORTC QLQ-STO22 and the FACT-Ga are site-specific questionnaires that are related to gastric cancer [20, 23]. Fifteen out of twenty-six studies have used the EORTC QLQ-C30 of which twelve studies also included the EORTC QLQ-STO22 module.

Only one validation study was identified, which assessed the use of the STO22 module in patients who were operated in curative or palliative setting. The module was found to have a good internal consistency (Crohnbach’s alpha’s >0.7) and was deemed reliable and sensitive to changes in both individual patient status and differences between patient groups [23].

Postoperative patients are considered a different entity in the DAUGS20 and Korenaga’s score, and these questionnaires focus specifically on patients following gastrectomy for cancer [11]. The questionnaires measure treatment-specific symptoms, such as appetite, swallowing, heartburn and diarrhoea [25, 26]. The Dysfunction After Upper Gastrointestinal Surgery (DAUGS20) questionnaire was originally designed in gastric and oesophageal cancer patients who had undergone surgery. The DAUGS is designed to measure QOL postoperative, and no baseline measurement is included [26]. An overview of the different PROMs is provided in Table 3.

## Discussion

The here-presented systematic review aimed to review what PROMs are available in assessing the QOL in patients with gastric cancer who undergo gastric resection. Ten PROMs were identified in 26 studies regarding different surgical techniques or comparison of different treatment modalities.

Gastrectomy with radical resection margins of 5 cm around the tumour along with adequate lymfadenectomy is currently the only curative therapy available in gastric cancer [27]. Overall QOL and even separate domains of QOL may differ between different treatment modalities. Question remains whether surgical patients should be considered a separate entity, and whether questionnaires should be developed or adapted for patients undergoing gastrectomy. In an optimal setting, the PROMs should allow for overall assessment of QOL, along with specific modules to assess specific effects associated with the disease and treatment [28].

The DAUGS20 and Korenaga’s score consider surgical patients to be a different entity. These questionnaires are specifically aimed at the postoperative patient who had surgery for gastric cancer [25, 29]. No validation studies regarding these questionnaires were available. DAUGS20 and Korenaga’s score are not developed for overall QOL assessment and are preferably to be used alongside a general QOL PROM [26, 30]. Since the questionnaires aim specifically at the postoperative patient, they do not allow for comparison of QOL among different treatment modalities such as chemotherapy and radiotherapy. They do allow for comparison of QOL among different surgical techniques.

The EORTC and FACT questionnaires consider gastric cancer patients as a whole. Both the EORTC and FACT questionnaires consist of a general cancer QOL module to which organ-specific module can be added (EORTC QLQ-STO22 and FACT-Ga), allowing for general and disease-specific QOL assessment between different treatment modalities. Both questionnaires were developed in patients with gastric cancer undergoing different treatment modalities, including surgery. With regard to comparability and reproducibility, the EORTC was used more often and might therefore allow for comparison to conducted studies, taking into account the heterogeneity in research questions, time points of QOL measurement and follow-up.

Fourteen (54 %) of the included studies consisted of retrospective cohort studies. Only six randomized studies were available. Differences in study design, endpoints, patient groups, surgical techniques and time points in the studies further limited assessment and pooling of data. No validation studies were available for the use of these PROMs in patients undergoing surgery for gastric cancer; hence, comparison of the performance of the different PROMs with regard to validity, internal consistency and discriminative ability was not possible.

Future research should focus on content validity of the used questionnaires in postgastrectomy patients in order to assess whether all the important domains are truly assessed and no items are missing. In order to further assess the use of PROMs in treatment of individual patients, our project group is currently aiming to develop a core outcome set of patient-reported outcomes in gastric cancer patients.

In conclusion, in the assessment of QOL in surgical gastric cancer patients, a great variety of PROMs are being used. A questionnaire with a general module to assess overall QOL, which can be supplemented with disease-specific modules allowing for the assessment or QOL of different treatment modalities, seems to be most desirable. With regard to current practice, the EORTC QLQ-C30 with STO22 module was developed in gastric cancer patients with different treatments, and it is used most widely, allowing for comparison of new data to studies that were already conducted. Future research should assess the need for treatment-specific modules.

## Appendix

**Table:**

Search	PubMed Query 17 November 2014	Items found
#9	Search #8 NOT (“addresses”[Publication Type] OR “biography”[Publication Type] OR “comment”[Publication Type] OR “directory”[Publication Type] OR “editorial”[Publication Type] OR “festschrift”[Publication Type] OR “interview”[Publication Type] OR “lectures”[Publication Type] OR “legal cases”[Publication Type] OR “legislation”[Publication Type] OR “letter”[Publication Type] OR “news”[Publication Type] OR “newspaper article”[Publication Type] OR “patient education handout”[Publication Type] OR “popular works”[Publication Type] OR “congresses”[Publication Type] OR “consensus development conference”[Publication Type] OR “consensus development conference, nih”[Publication Type] OR “practice guideline”[Publication Type]) NOT (animals[mh] NOT humans[mh])	1610
#8	Search #5 AND #6 AND #7	1625
#7	Search (HR-PRO[tiab] OR HRPRO[tiab] OR HRQL[tiab] OR HRQoL[tiab] OR QL[tiab] OR QoL[tiab] OR quality of life[tiab] OR life quality[tiab] OR health index*[tiab] OR health indices[tiab] OR health profile*[tiab] OR health status[tw] OR ((patient[tiab] OR self[tiab] OR child[tiab] OR parent[tiab] OR carer[tiab] OR proxy[tiab]) AND ((report[tiab] OR reported[tiab] OR reporting[tiab]) OR (rated[tiab] OR rating[tiab] OR ratings[tiab]) OR based[tiab] OR (assessed[tiab] OR assessment[tiab] OR assessments[tiab]))) OR ((disability[tiab] OR function[tiab] OR functional[tiab] OR functions[tiab] OR subjective[tiab] OR utility[tiab] OR utilities[tiab] OR wellbeing[tiab] OR well being[tiab]) AND (outcome[tiab] OR outcomes[tiab] OR index[tiab] OR indices[tiab] OR instrument[tiab] OR instruments[tiab] OR measure[tiab] OR measures[tiab] OR questionnaire[tiab] OR questionnaires[tiab] OR profile[tiab] OR profiles[tiab] OR scale[tiab] OR scales[tiab] OR score[tiab] OR scores[tiab] OR status[tiab] OR survey[tiab] OR surveys[tiab])))	1657339
#6	Search “gastrectomy”[MeSH] OR ((gastrectom*[tiab] OR “gastric resection”[tiab] OR gastrectom*[ot] OR “gastric resection”[ot]) NOT medline[sb])	27902
#5	Search “Stomach Neoplasms”[Mesh] OR Stomach Neoplasm*[tiab] OR Gastric Neoplasm*[tiab] OR Stomach Cancer*[tiab] OR Gastric Cancer*[tiab] OR Stomach carcinoma*[tiab] OR gastric carcinoma*[tiab] OR stomach tumor*[tiab] OR stomach tumour*[tiab] OR gastric tumor*[tiab] OR gastric tumour*[tiab] OR stomach neoplasia*[tiab] OR cardia carcinoma*[tiab] OR linitis plastica[tiab] OR Stomach Neoplasm*[ot] OR Gastric Neoplasm*[ot] OR Stomach Cancer*[ot] OR Gastric Cancer*[ot] OR Stomach carcinoma*[ot] OR gastric carcinoma*[ot] OR stomach tumor*[ot] OR stomach tumour*[ot] OR gastric tumor*[ot] OR gastric tumour*[ot] OR stomach neoplasia*[ot] OR cardia carcinoma*[ot] OR linitis plastica[ot]	87062
